# The Impact of Underlying Conditions on Quality-of-Life Measurement Among Patients with Chronic Wounds, as Measured by Utility Values: A Review with an Additional Study

**DOI:** 10.1089/wound.2023.0098

**Published:** 2023-10-19

**Authors:** Kristen A. Eckert, Caroline E. Fife, Marissa J. Carter

**Affiliations:** ^1^ Strategic Solutions, Inc., Bozeman, Montana, USA.; ^2^ Intellicure, LLC, The Woodlands, Texas, USA.; ^3^ U.S. Wound Registry (501 3C Nonprofit), The Woodlands, Texas, USA.; ^4^ Baylor College of Medicine, Houston, Texas, USA.

**Keywords:** wounds, comorbidities, multimorbidities, utility values, Wound-Quality-of-Life questionnaire, Wound-QoL

## Abstract

**Significance:**

Quality of life (QoL) is important to patients with chronic wounds and is rarely formally evaluated. Understanding what comorbidities most affect the individual versus their wounds could be a key metric.

**Recent Advances:**

The last 20 years have seen substantial advances in QoL instruments and conversion of patient data to a single value known as the health utilities index (HUI). We review these advances, along with wound-related QoL, and analyze real-world comorbidities challenging wound care.

**Critical Issues:**

To understand the impact of underlying comorbidities in a real-world patient population, we examined a convenience sample of 382 patients seen at a hospital-based outpatient wound center. This quality reporting study falls outside the regulations that govern human subject research. Comorbid conditions were used to calculate HUIs using a variety of literature-reported approaches, while Wound-Quality-of-Life (W-QoL) questionnaire data were collected from patients during their first visit. The mean number of conditions per patient was 8; 229 patients (59.9%) had utility values for comorbidities/conditions, which were worse/lower than their wounds' values. Sixty-three (16.5%) patients had depression and/or anxiety, 64 (16.8%) had morbid obesity, and 204 (53.4%) had gait and mobility disorders, all of which could have affected W-QoL scoring. The mean minimum utility value (0.5) was within 0.05 units of an average of 13 studies reporting health utilities from wound care populations using the EuroQol 5 Dimension instrument.

**Future Directions:**

The comorbidity associated with the lowest utility value is what might most influence the QoL of patients with chronic wounds. This finding needs further investigation.

**Figure:**
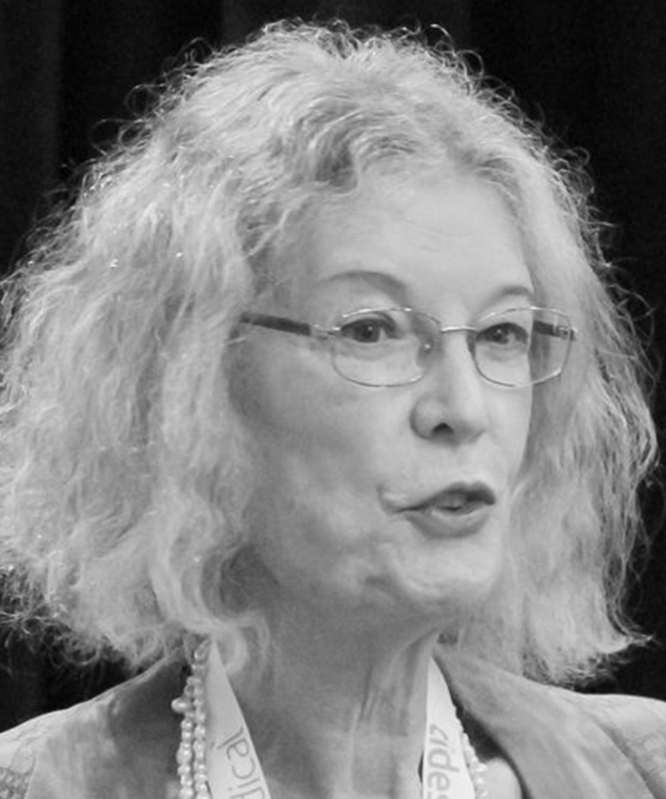
Marissa J. Carter, PhD, MA

## SCOPE AND SIGNIFICANCE

Quality of life (QoL) has become a cornerstone in understanding how the patient feels about treatment and outcomes for medical conditions. While general health-related quality-of-life (HR-QoL) instruments are sometimes used in controlled and real-world clinical wound care studies, they do not inform clinicians how the patient feels about the healing or lack of healing of chronic wounds. To address that issue, several wound-specific QoL questionnaires have been developed. Another concept of QoL is the utility value, which is represented on a scale of 0 (the state of death) to 1 (perfect health).

By the late 1980s, utility values were gaining traction in QoL research,^1^ and since then, several methods have been validated to elicit values from patients with a single disease, exploring dimensions and severity, using direct interactive processes. In addition, utility values from patients with unique health profiles have been obtained from HR-QoLs, such as the EuroQol 5 Dimension (EQ-5D), to convert each health profile value by applying a set of weights specific to a given country.^2^

## TRANSLATIONAL RELEVANCE

These QoL approaches have been helpful in characterizing the QoL of patients with chronic wounds, but we still lack a deep comprehension of how unhealed chronic wounds affect the typical patient with many serious comorbidities that compete for attention. Moreover, the way that individuals value QoL, as exemplified by the European Quality of Life 5 Dimensions 5 Level Version (EQ-5D-5L), varies substantially between countries with the largest differences, comprising one-third of the QoL value.^3^ This is troubling in of itself, but it also affects the impact assessment of chronic wounds in different populations.

## CLINICAL RELEVANCE

The ultimate goal of wound care physicians is to heal chronic wounds. However, since chronic wounds are almost always a symptom of underlying comorbid disease(s), clinicians must approach patient care holistically by addressing the impact of these diseases on the wound healing process. Whether impaired mobility, social distress, pain, depression, and restricted lifestyle contribute to the development of a chronic wound or are the result of them, these underlying conditions significantly lower QoL.^4^ Not only can these problems, such as depression, possibly reduce wound healing (perhaps through psychoimmunological effects) but also pain, immobility, and older age can override the wound status (healed or active) as the driver for QoL,^5^ rendering the use of wound-related QoL instruments inappropriate in clinical practice.

## BACKGROUND

Our aims in this review were to (1) inform readers how we measure QoL in patients, including wound-related QoL instruments and utility values; and (2) explain the problems of evaluating QoL in patients with chronic wounds *and* serious comorbidities, illustrated by a detailed analysis of comorbidities and associated utility values in a relatively large cohort of real-world patients, who had the Wound-QoL (W-QoL) questionnaire administered before wound care.

### Literature search for utility values

The Cost-Effectiveness Analysis Registry (CEAR) at the Center for the Evaluation of Value and Risk in Health Tufts Medical Center was searched for all relevant utility values related to patient comorbidities and wounds under the “Utilities” tab.^6^ A separate PubMed literature search of any newer utility value for wounds and ulcers only was conducted for articles published from 2020 through March 2023. The search terms used were “wounds,” “ulcer,” “utility values,” and “utilities.”

### Prevalence and costs of chronic wounds

The global prevalence of chronic wounds is estimated at 1.51 to 2.21 per 1,000 population.^7^ In the United States, chronic wounds affect about 16.4% of the population on Medicare, with total annual costs ranging from U.S.$ 22.5 to $67.1 billion.^8^ The most common wound type among patients older than 65 years is the dehisced surgical wound, occurring in up to 30% of patients;^9^ it can result in mortality rates as high as 50%.^10,11^

Despite the national focus on diabetic foot ulcers (DFUs), venous leg ulcers (VLUs), and pressure injuries (PIs), the most common chronic ulcer is “nameless” and can be directly linked to the patient's underlying medical problem(s). As symptoms of underlying disease, it is not surprising that chronic wounds are often recurrent, with up to 40% of DFUs and 69% of VLUs recurring within 1 year.^12,13^ Deep (stage 3 or 4) PIs can also be among the most challenging to heal, because the volume of the open wound can be very large.^14^

There has long been interest in using QoL among patients with chronic wounds to evaluate the therapeutic benefit of various treatments separate from or in conjunction with actual wound healing. For example, arterial ulcers, although less prevalent than other chronic wounds, are associated with the worst health state due to causing more pain, anxiety/depression, greater sleep disturbance, and restricted mobility and self-care.^4,15–17^ Patients may perceive an improvement in these factors to be even more important than healing the wound. Because healing may not always be possible, there is significant interest in identifying QoL factors that wound management can positively impact.

### HR-QoL measures

In the last 47 years, the estimated number of studies published yearly with the keyword “quality-of-life” in PubMed grew to over 17,000,^19^ and this is probably a considerable underestimate. That the patient is finally being recognized as the center of the medical universe is an important landmark. However, it also means that clinicians have to choose among a plethora of instruments and questionnaires; unfortunately, not all HR-QoL measures may be interchangeable.^18^

In general, HR-QoL refers to how a person feels about physical functioning, including mobility, ability to dress and bathe, and role functioning, which includes being productive in work, capable of caring for oneself, and having a role in immediate and extended social networks. Those elements are most influenced by health conditions, treatment,^19^ and well-being, which is summation of such states as happiness, sadness, depression or anxiety, pain, and lethargy.

From the mid-1970s through the 1990s, the short form (SF)-12 and SF-36 QoL instruments were developed,^20–22^ with the latter translated into health state utilities.^23^ In essence, this was the SF-6D, an econometric preference-based index derived from 11 items of the SF-36, which are combined into 6 dimensions of health of 4–6 levels each.^24^ In Europe, the EQ-5D was created in roughly the same time frame as the SF-36 and went through several minor revisions regarding ceiling effects and lack of sensitivity, as well as language translations.^25^ The newest version, the EQ-5D-5L, has 3,125 health states (5 dimensions of 5 levels).^26^ This version is considered to be the most popular of the so-called multiattribute utility instruments (MAUIs).^27^

Utility values fall into the category of preference-based measures (PBMs) in which a single numeric metric is used to “value” a given health state. Because these measures incorporate weights that reflect the importance attached to a set of specific health aspects, PBMs reflect the overall quality of an individual patient's (perceived) health status.^28^ The issue that affects the interpretability of utility values derived from MAUIs to date is the mapping process to a given population, which is subject to errors and biases.

While there are several aspects to mapping processes required to properly address a national population, the basic idea is to take an appropriately sized sample, analyze the demographic attributes between EQ-5D-5L scores,^28^ and subsequently ask each participant to evaluate a number of given health states using both composite time trade-off (cTTO) and discrete choice experiment (DCE) approaches (see next section for discussion on methods to elicit utility values). By example, the value for health state 23245 (mobility: slight; self-care: moderate; usual activities slight; pain/discomfort: severe; and anxiety/depression: severe) is calculated as follows: 1 − (0.058 + 0.080 + 0.050 + 0.276 + 0.289) = 0.247. Each of these individual disutility values was obtained by multiplying the raw subject-derived scores by the weighted averages of slopes for the three latent groups correcting for subject heterogeneity regarding the relationships between disutility and health state severity in a Bayesian regression model.^29^

Patient-reported outcome measures (PROMs) have become increasingly common in the last 15 years, but they are not PBMs, because they lack an associated, preference-based scoring system. However, if another dataset has the same outcomes that are measured in the relevant clinical study/studies, as well as the patient's responses to a standard PBM, then this external dataset can be used to estimate a statistical relationship between the two types of outcome measure.^30^

This permits outcome data from the trial to create an estimate on the effect of the treatment in health utility terms.^29^ This form of mapping utilizes both the direct approach, in which utilities are estimated directly from explanatory variables, and the indirect approach (response mapping), which first predicts the probabilities for each response to each MAUI question, and then uses relevant weights to convert them into utilities.^31^ The statistical techniques used are becoming more complex, including the use of machine learning, but they are yielding better results.

### Methods of directly obtaining utilities

Historically, the concept of utility has evolved from one based on game theory to patient-based preferences about health states.^32,33^ The key vehicle in which this can be better understood is the standard gamble (SG). In this study, an individual makes a series of hypothetical choices between continuing life in the current state of health or gambling to achieve a state of perfect health or death:

The subject is offered two alternatives. Alternative 1 is a treatment with two possible outcomes: either the patient is returned to normal health and lives for an additional T years (probability *p*), or the patient dies immediately (probability 1 − *p*). Alternative 2 has the certain outcome of chronic state i for life (T years). Probability *p* is varied until the respondent is indifferent between the two alternatives, at which point the required preference value for state i (using the above notation) is simply *p*.^34^

Another approach developed to mitigate some of the SG shortcomings is time trade-off (TTO).^33^ In its simplest form, the TTO task requires respondents to trade-off a portion of remaining life in relation to health status by iteratively choosing a shorter life in full health (*i.e*., a utility value of 1) against a longer time experiencing the disutility of the current health state until a point of indifference is reached.

One problem with this method is that the constant proportional TTO assumption is frequently violated, which means that patient preferences are not properly captured.^34^ Another is the problem of valuing health states considered to be the “worse than dead,” resulting in extremely negative values, which led to the development of the cTTO (composite) method. For those health states in which all time was traded away, the idea of lead time was introduced, which allows the participant to move between negative and positive values without being required explicitly to think about whether the state is worse or better than being dead.

Because it does not require a separate method of elicitation for these states, it avoids the “focusing effect” that may arise with such deliberation.^35^ However, it is also becoming clear that new variants of the TTO approach have not completely solved many issues^36,37^—(one reason why DCEs are also employed). Unlike TTO, DCE requires respondents to choose the one they prefer in pairwise health state comparisons, instead of making participants go through an iterative process of identifying the indifference point between choices.^38,39^

No particular approach is without its detractions, which is why so much research continues to be conducted in the field. Part of the goals is to understand why individuals struggle to value their lives and sometimes the irrationality of their choices and develop instruments that account for these problems.

### Wound-related PROMs

MAUIs do not capture many dimensions of wounds that are important to patients, such as odor or exudate levels. Consequently, in the last two decades, considerable efforts have been devoted to the development of wound-specific PROMs. Nevertheless, how useful are they? Authors of a recent systematic review studied 33 such PROMs, and evaluated the quality of their properties using the Consensus-based Standards for the Selection of Health Measurement Instruments guidelines.^40-42^ The results were worrying. In terms of PROM design score, only 1 rated as adequate and only 2 scored very good (the Spinal Cord Injury-QOL and WOUND-Q). In the cognitive interview/pilot test scores, 31 scored less than adequate, and 2 were not scored. Regarding overall quality of development rating, no study had an adequate or very good rating. This should prompt the question, how valid are PROMs?

Since a complete assessment of wound-specific PROMs is beyond the scope of this review, and the W-QoL was the initial focus of our study, it is used herein as an illustration in terms of development and issues. Previously validated wound QoL instruments, such as the Cardiff Wound Impact Schedule, Freiburg Life Quality Assessment, and Würzburg Wound Score have been criticized by patients for requiring too much effort to use, and so, researchers in Germany sought to create a shorter and more practical questionnaire that still encompassed the core components of the three previously validated instruments. They developed the W-QoL with 17 questions classified into 3 subscales: everyday life, body, and psyche.^43^

Each question is scored on a 5-point Likert scale from 0 (not at all) to 4 (very much), and a total score is generated. The W-QoL was tested in the United States using a dataset from 388 patients at a hospital-based outpatient wound center.^44^ Item response theory analyses determined that the instrument could be further refined after being unable to confirm the factor structure underlying the model. In addition, some patients had difficulty answering some questions, such as those related to climbing stairs and feeling dependent on others. For example, patients who already experienced mobility issues, such as those who were in a wheelchair or amputees, were already codependent on others, and were unable to move freely and/or climb up the stairs.^44^

Using a global dataset of 1,185 patients from the United States, Germany, Spain, the Netherlands, Sweden, and Israel, the W-QoL was simplified to only 14 questions, after removing the questions related to hitting the wound against something, climbing stairs, and the financial burden of the wound.^45^ Since then, the W-QoL has shown to be useful for assessing QoL in acute blast or gunshot wounds.^46^ The revised W-QoL questionnaire still does not address the influence of preexisting comorbidities and conditions that could already limit or impact a patient's mental health, independence, and pain levels.^45^ Therefore, additional analysis of the effect of comorbidities on W-QoL scores is warranted.

### Effect of wound comorbidities on QoL in wound care

A major limitation to assessing QoL in patients with wounds is that, while instruments specifically target the burden of the wound, chronic wounds are most often a result of underlying conditions and chronic comorbidities that can already drastically reduce overall HR-QoL. Authors have called arthritis, back pain, and depression the most influential comorbidities on QoL,^47^ and these three comorbidities influence a patient's level of pain, movement and daily activities, and mental health, all of which are captured by the three subscales of the W-QoL.^45^ The effect of multimorbidity, when a patient has two or more chronic diseases, on QoL is not captured by PROMs.^48^ Multimorbidities are especially prevalent in the population with diabetes (among more than a quarter of patients) and are known to significantly decrease QoL,^49,50^ and without comprehensive tools to assess their impact, it appears that they may negatively impact treatment effectiveness.^48,51^

Up to 90% of the elderly population may have at least one comorbidity, with cardiovascular disease, neurological disease, diabetes, and malnutrition being associated with the development of PIs in these patients.^52,53^ It is also disconcerting that trends from the past decade demonstrate an increasing prevalence of chronic wounds among Medicare beneficiaries younger than 65 years, a patient population that is only eligible for Medicare due to having a disability, which could be caused by an underlying comorbidity that impairs wound healing.^8^ The inability of PROMs and wound QoL assessments to address the impact of comorbidities on patient QoL is a major limitation in wound care research, but it is possible to analyze the impact of comorbidities on W-QoL scores, by analyzing utility values associated with comorbidities and their association with W-QoL scores.

### Methods

This study was a secondary analysis of data from a representative, convenience sample of sequential patients presenting at a hospital-based outpatient wound center from June 26, 2014, through March 10, 2016, which was previously analyzed to assess the use of the W-QoL questionnaire in the United States.^44^ An electronic laboratory platform was not used for data collection; instead, direct-from-EHR (electronic health record) data were used for the purpose of testing a new quality measure evaluating the use of the W-QoL questionnaire.

The U.S. Wound Registry (USWR) is recognized by The Centers for Medicare and Medicaid Services (CMS) as a qualified clinical data registry, which is empowered by CMS to develop specialty-specific quality measures.^44^ At the time of this project, CMS endorsed the W-QoL as a quality measure for reporting to the Quality Payment Program and considered it a practice improvement activity for physicians.^44^ As such, informed consent was not required, and The Woodlands Institutional Review Board (IRB, The Woodlands, TX) provided IRB exemption of this study as a quality improvement initiative, which fell outside the regulations that govern human subject research. However, this study still adhered to the Declaration of Helsinki. All patients signed a release for photographs for academic purposes.

The convenience sample comprised all patients able to answer the W-QoL questionnaire using an electronic tablet during their first visit. Patients unable to fill out the questionnaire due to a medical condition, such as moderate to advanced dementia, or a language barrier were excluded from this analysis. Patients with mild dementia/Alzheimers were included. The electronic tablets were programmed for the W-QoL, so that patients could answer the questionnaire separate from the data captured at point of care by the EHR. The nurse handed each patient the electronic tablet to fill out, which was done without staff interaction, with instructions to inform the nurse when they finished the questionnaire.

**Figure 1.**
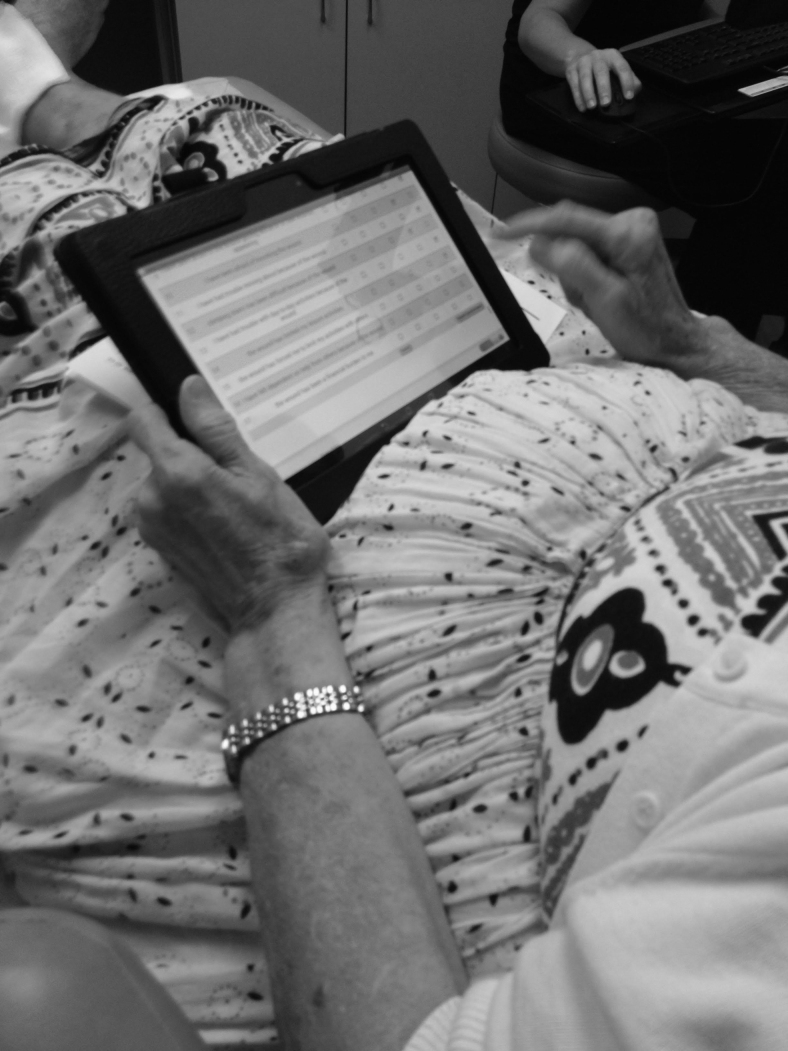
An elderly patient with rheumatoid arthritis uses an electronic tablet provided by her provider to fill out the Wound-Quality-of-Life questionnaire. Her only complaint at the time was that the inexpensive tablet used for this analysis did not have as good as resolution as her personal tablet at home.

The original W-QoL questionnaire with 17 items was used by patients, although several questions were modified so that the English translation was clear (Box 1). The W-QoL data were then transmitted electronically to the USWR, which later merged those data with the EHR wound data for each patient record. Figure 1 depicts an elderly patient from this convenience sample with rheumatoid arthritis using a tablet unaided to fill out the W-QoL. All patients were able to complete the W-QoL questionnaire. There were 388 patients with wounds included in the original dataset; only 6 (1.5%) patients had no comorbidity and were excluded from the analysis, leaving 382 patients to be analyzed.

The USWR generated a list of wounds and comorbidities for these patients and provided deidentified data for analysis. The utility values for all comorbidities and wounds were searched on the CEAR. Generally, the database search engine returned the most relevant references first. For heart failure, the CEAR retrieved 485 articles that sourced potentially relevant utility values. However, only the first 100 utility values registered were screened for potential inclusion in this analysis, because they were the most relevant.

After the first 100 entries, values were very specific to multimorbidities that could not be generalized or were redundant. Likewise, for multiple sclerosis, there were over 300 articles retrieved. The first 200 were screened for potential inclusion, because they were the most relevant. Unique utility value data were collected in an Excel spreadsheet; duplicate values were excluded, unless the condition descriptor changed (*e.g*., the same utility value may have been calculated for mild vs. moderate disease and was counted separately). The median utility value was calculated for the purposes of the assigned utility value to each comorbidity that a patient had. The comorbid conditions with the 10 worst/lowest utility values were determined and their frequency calculated. The frequency of patients whose comorbidities had lower/worse values than their wounds was also calculated.

There were 33 conditions that did not have any utility value reported in the CEAR (Supplementary Table S1). These conditions were excluded from analysis. There were 81 conditions that did not have utility values reported in the CEAR, but proxy utility values for similar conditions were imputed (Supplementary Table S2). Supplementary Table S3 lists the 352 comorbidities and wound types analyzed in this study with their median utility values. Patients with paraplegia used the utility value for wheelchair bound (0.35), unless they separately had listed that they were bedridden/had bed confinement status (0.13).

Note that a median utility value for a DFU was 0.65, but if at least two comorbidities were present, it decreased to 0.49. The utility values collected for surgical wounds did not include surgical site infections (SSIs), as the comorbidities listed in this analysis did not specify that patients had SSIs. Burns did not have utility values reported, but third-degree burns had deep necrosis noted, so that value was imputed as a proxy, while all other burns were valued as a complicated wound (Supplementary Tables S2 and S3). Complicated, nonhealing wounds were valued at 0.61 and imputed for complex wounds that did not have specific values for their wound type. An uncomplicated open wound was 0.7 and used for lacerations/accidents and other wounds that were not complex or chronic. Supplementary Table S4 lists the utility values, descriptors used, and reference sources for all wounds.

Major amputations included amputation of limbs, below-knee amputations (BKA), and above-knee amputations. Minor amputations included amputations of toes. In Supplementary Tables S3 and S4, it is noteworthy that a major amputation with diabetes had a median utility value of 0.61, whereas a major amputation without diabetes was much lower at 0.4; this difference was due to the fact that there were some high values for diabetes with BKA/major amputation reported at 0.8 (using the TTO approach and other indirect/generic measures), which is likely due to the fact that the patient's suffering was much worse before the amputation than after the event (Supplementary Table S4). However, any complication to the amputation greatly lowered the value.

To further understand how the presence of a particular comorbidity could potentially affect the patient's response to the W-QoL questionnaire, relevant W-QoL questions were selected and grouped together. Only four questions from the original questionnaire could be considered exclusive to wound status: No. 2 referring to wound smell, No. 3 referring to wound discharge, No. 5 referring to wound treatment burden, and No. 17. referring to the financial burden caused by the wound (Box 1). Several years after patient data were collected for this analysis, the original authors of the W-QoL refined and simplified the questionnaire, removing Nos. 10, 12, and 17.^45^ Thus, for our analysis, we excluded Nos. 2, 3, 5, 10, 12, and 17. The remaining questions were grouped by pain (No. 1), emotions and mental health (Nos. 6–9), sleep disturbance (No. 4), and limited activities, movement, and independence (Nos. 11, 13–16).

The number of patients with pain and mental health disorders, gait and mobility disturbances, and conditions that could cause sleep disturbances were counted and tabulated. All gait and mobility disturbances, paralysis, and bedridden status were counted, including leg muscle contractures, myotonic dystrophy, transverse myelitis, atherosclerosis of native arteries of the extremities with ulceration, diabetic neuropathy, chronic obstructive pulmonary disease, slipping, tripping, and stumbling without falling, morbid obesity, heart failure, multiple back surgeries causing gait and mobility disturbances, Charcot deformity, major amputations, amputation of the big toe, spinal cord injury, paraplegia, quadriplegia, functional quadriplegia, and postpolio paralysis. Morbid obesity was also counted separately.

Descriptive statistics were used to summarize patient demographic and clinical characteristics. The lowest utility value recorded per patient was analyzed to determine if it was worse than patients' wound values. The subset of patients with comorbidities worse than wounds was counted.

For the utility value analysis, there were 382 patients with 3,040 comorbidities in the final dataset, of which 63 had no associated utility value. These cases were deleted, leaving 382 patients with 2,997 comorbidities. Mean, median, minimum, and maximum utility values for each patient were calculated using descriptive statistics (SPSS [PASW 28]; IBM, Armonk, NY). Utility values derived using standard additive, multiplicative, and adjusted decrement estimator (ADE) approaches for each patient's set of comorbidities were derived using the methods of Thompson et al.^99^ (the linear index method could not be employed, as the number of possible comorbidity combinations was too high) (Box 2).

We then compared our estimates of health utilities to those derived from the EQ-5D reported in the literature for chronic wound populations.

### Results of analysis

All 382 patients (46.4% female, 172/369; 89.3% White, 341/378) answered the W-QoL questionnaire during their first visit. Table 1 summarizes patients' demographics and clinical characteristics. Their mean (standard deviation [SD]) age was 64 years (16.4). The patients had nine different wound types; the three most common wounds were VLUs occurring in 23.6% of patients (*n* = 90), surgical wounds occurring in 18.6% (*n* = 71), and PIs in 12.6% (*n* = 48).

**Table 1.** Patient demographic and clinical characteristics (*n* = 382)

	No. of Patients (%)
Sex^a^ (*n* = 369)	
Male	197 (53.4)
Female	172 (46.6)
Race/Ethnicity^b^ (*n* = 378)	
White	341 (89.3)
Black	12 (3.1)
Asian	4 (1)
Hispanic	24 (6.3)
Other	13 (3.4)
Unknown	12 (3.1)
Wound types	
VLU	90 (23.6)
Surgical wound/Dehiscence^c^	71 (18.6)
PI	48 (12.6)
DFU	44 (11.5)
Other, complicated wound	31 (8.1)
Arterial ulcer	17 (4.5)
Burn	13 (3.4)
Pyoderma gangrenosum	8 (2.1)
Uncomplicated acute wound	8 (2.1)
Most common comorbidities	
Hypertension	255 (66.8)
Any gait and mobility disorders	204 (53.4)
Edema	135 (35.3)
Diabetes	141 (36.9)
Chronic pain	129 (33.8)
Hyperlipidemia/pure hypercholesterolemia	110 (28.8)
Hypothyroidism	92 (24.1)
Any neuropathy	73 (19.1)
Obesity (not morbid)	68 (17.8)
Venous insufficiency	66 (17.3)
Level of activity	
Active	154 (40.3)
Minimally active	101 (26.4)
Temporarily restricted	56 (14.7)
Sedentary	40 (10.5)
Unknown	31 (8.1)
Alcohol status	
Does not drink alcohol	134 (35.1)
Unknown	106 (27.7)
Smoking status	
Current smoker	37 (9.7)
Former smoker	133 (34.8)
Never smoked	198 (51.8)
Unknown	14 (3.7)

^a^ Data were available for 369 patients.

^b^ Data were available for 378 patients.

^c^ Includes 4 patients with pilonidal cysts that were surgically drained and became nonhealing surgical wounds and 19 patients with active wounds from amputations; excludes patients with DFUs on stumps.

DFU, diabetic foot ulcer; PI, pressure injury; VLU, venous leg ulcer.

The mean (SD; range) number of comorbidities/conditions per patient was 8 (3.9; 2 to 26). The three most common conditions were hypertension (66.8%, *n* = 255), gait and mobility disorders (53.4%, *n* = 204), and edema (35.3%; *n* = 135) (Table 1). Table 2 lists the 10 worst conditions, which have the lowest utility values. Notably, 30 patients (7.9%) were bedridden, which was the worst utility value at 0.13. After proxy utility values were imputed for conditions with missing values (Supplementary Tables S1 and S2), 52 patients (13.6%) still had missing utility values for their conditions. The mean (SD; range) number of missing values per patient was 0.2 (0.4; 0 to 3). The mean (SD; range) percentage of conditions with missing values per patient was 1.8% (5.2; 0% to 33.3%). Importantly, 229 patients (59.9%) had utility values for conditions that were worse/lower than their wound utility values.

**Box 1.** Modified Wound-Quality-of-Life questionnaire

Item No.	Occurring in the Last 7 Days
1	… my wound hurt
2^a^	… my wound had a bad smell
3^a^	… my there was a disturbing discharge from the wound
4	… the wound has affected my sleep
5^a^	… the treatment of the wound has been a burden to me
6	… the wound has made me unhappy
7	… I have felt frustrated because the wound is taking so long to heal
8	… I have felt worried about my wound
9	… I have been afraid of the wound getting worse or of new wounds appearing
10^b^	… I have been afraid of hitting the wound against something
11	… I have had trouble moving around because of the wound
12^b^	… climbing stairs has been difficult because of the wound
13	… I have had trouble with everyday activities because of the wound
14	… the wound has limited my leisure activities
15	… the wound has forced me to limit my activities with others
16	… I have felt dependent on help from others because of the wound
17^a,b^	… the wound has been a financial burden to me

^a^ Excluded from analysis, because the question is wound-specific and likely would not have been influenced by other comorbidities.

^b^ Excluded from analysis, because the questionnaire was later refined and simplified to ease patient use.^46^

**Box 2.** Simple explanation of the utility calculation methods

Method	Simple Explanation
Minimum	The lowest value in a utility value set of comorbidities
Additive	The sum of the disutilities measured in populations with only the individual comorbid conditions
Multiplicative	Disutility impact is proportional to the disutility already experienced; for example, A × B × C, where A, B, and C are the utility values associated with health conditions A, B, and C
ADE	The upper limit for the utility value for any joint health condition (*e.g*., health conditions A and B) is set at the minimum for any single health state from the set of health state utility values. Each additional health state utility value is a function of the health state utility values patients already have, adjusted by the minimum value.

For explicit definitions and detailed mathematical equations see the supplementary materials included in the Thompson et al.^100^ article. Disutility = 1 − utility; for example, if utility value for diabetes is 0.9, disutility would be 0.1.

ADE, adjusted decrement estimator.

**Table 2.** The 10 worst conditions in ascending order from lowest utility value

Comorbidity	Utility Value	No. of Patients (%)
Bedridden	0.13	30 (7.9)
Chronic liver disease	0.22	1 (0.3)
Kyphoplasty	0.28	1 (0.3)
Generalized weakness	0.29	1 (0.3)
Wheelchair bound	0.35	2 (0.5)
Polio	0.37	2 (0.5)
Monoplegia/paralysis	0.37	1 (0.3)
Quadriplegia	0.37	6 (1.6)
Major amputation without diabetes	0.4	3 (0.8)
Flap	0.4	1 (0.3)

Table 3 summarizes the frequency of burden of comorbidities that could have potentially influenced a patient's response to the W-QoL questions. Ten (71.4%) of the refined W-QoL questions could have potentially been influenced by patients' preexisting conditions, that is to say, the patient could have reported a lower W-QoL score, because their comorbidities were already painful and/or a burden on their mental health, emotions/feelings, and activities of daily living. For example, a third of patients (*n* = 129) reported chronic pain, which would have caused them pain, sleep disturbance, and limited their daily activities, movement, and independence, potentially influencing their response to seven W-QoL questions.

**Table 3.** Summary of analysis of Wound-Quality-of-Life questions whose response could potentially be influenced by patients' preexisting comorbidities and conditions

Comorbidities That Could Have Decreased QoL	No. of Patients (%)	W-QoL Variable(s) Affected by Comorbidity (Relevant Questionnaire Numbers)
Chronic pain	129 (33.8)	Pain (1) Sleep disturbance (4) Limited Daily Activities, Movement, and Independence (11, 13–16)
Arthritis, any type	64 (16.8)	Pain (1) Sleep disturbance (4) Limited Daily Activities, Movement, and Independence (11, 13–16)
Depression/anxiety^a,b^	63 (16.5%)	Pain (1) Emotions and mental health (6–9) Sleep disturbance (4) Limited Daily Activities, Movement, and Independence (11, 13–16)
PTSD	4 (1%)	Pain (1) Emotions and mental health (6–9) Sleep disturbance (4) Limited Daily Activities, Movement, and Independence (11, 13–16)
Obesity/Morbid obesity	68 (17.8)	Sleep disturbance (4)
Heart failure	35 (9.2)	Sleep disturbance (4)
Apnea	32 (8.4)	Sleep disturbance (4)
Edema	135 (35.3)	Sleep disturbance (4)
Uterine cancer (active)	1 (0.3)	Sleep disturbance (4)
COPD	24 (6.3)	Sleep disturbance (4)
GERD	12 (3.1)	Sleep disturbance (4)
Urinary/bowel incontinence	17 (4.4)	Sleep disturbance (4)
Restless legs	6 (1.6)	Sleep disturbance (4)
Morbid obesity	64 (16.8)	Limited Daily Activities, Movement, and Independence (11, 13–16)
Gait and mobility disorder(s)^c^	204 (53.4)	Limited Daily Activities, Movement, and Independence (11, 13–16)

^a^ Nine (2.4%) had anxiety.

^b^ Fifty-six (14.7%) had depression.

^c^ Thirty (7.9%) were bedridden and an additional 20 (5.4%) had paralysis, paraplegia, quadriplegia, spinal cord injury, and/or were in a wheelchair.

COPD, chronic obstructive pulmonary disease; GERD, gastroesophageal reflux disease; PTSD, posttraumatic stress disorder; QoL, quality of life; W-QoL, Wound-Quality-of-Life.

More than half of patients (*n* = 204, 53.4%) had specific gait and mobility disorders, which would already have made movements difficult; 20 (5.4%) had paralysis, paraplegia, quadriplegia, spinal cord injury, and/or were in wheelchair; 30 (7.9%) were bedridden; and 64 (16.8%) were morbidly obese. Sixty-three (16.5%) patients had depression and/or anxiety. More than one-third of patients (*n* = 135) likely had sleep disturbances due to edema.

The utility values obtained by standard literature methods (Table 4) showed a wide variety with mean values for the minimum, additive, multiplicative, and ADE approaches of 0.497, −1.138, 0.129, and 0.217, respectively. Differences between means and median values for these standard approaches were minimal, while ranges (minimum to maximum) were largest for the additive approach, and floor effects were observed for the multiplicative and ADE approaches.

**Table 4.** Utility value metrics derived from 382 patients and their associated comorbidities

Utility Method	Mean^a^	Median^a^	Range^a^
Utility value (mean)^b^	0.718	0.726	0.425 to 0.871
Utility value (median)^b^	0.733	0.756	0.425 to 0.950
Utility value (maximum)^b^	0.890	0.900	0.425 to 0.996
Utility value (minimum)	0.497	0.520	0.131 to 0.735
Utility value (additive)	−1.138	−1.022	−5.148 to 0.585
Utility value (multiplicative)	0.129	0.0831	0 to 0.619
Utility value (ADE)	0.217	0.186	0.001 to 0.701

^a^ The mean, median, and range represent utility values derived from all patients in the dataset for the particular method used.

^b^ The utility value mean, median, and maximum of the utility methods refer to standard methods of calculating the means, median, or largest value from a set of utility values for a single patient.

ADE, adjusted decrement estimator.

## DISCUSSION OF FINDINGS AND RELEVANT LITERATURE

In this analysis of the impact of comorbidities on wound QoL, the patient population had predominantly chronic and/or complex wounds, which were difficult to heal; only 2.1% of patients had uncomplicated acute wounds. Unsurprisingly, this real-world patient population, typical of individuals with chronic wounds, was very sick, with patients having, on average, eight comorbidities/conditions each. More than two-thirds of the patients had hypertension; more than half had gait and mobility disorders; and more than one-third had diabetes and/or chronic pain (Table 1).

Compared to previous, larger studies of wound comorbidities,^52,100^ we analyzed comorbidities/conditions within the context of the W-QoL domains, and so general conditions related to ambulatory status (*i.e*., being bedridden, in a wheelchair, *etc.*) were captured, which were not necessarily reported by other authors. Our smaller population of 382 patients had twice as many comorbidities, on average, compared to an analysis of 3,000 patients treated for wounds in 2017 and 2018 in the National Health Service of the United Kingdom (UK).^52^

Those authors reported that patients had an average of 4.1 comorbidities, and more than half (57%) had diabetes, the presence of which was associated with delayed wound healing due to other coexisting multimorbidities, including cardiovascular disease (59%), renal disease (19%), and immunological disorders (11%). It is not clear how many wounds were chronic versus acute in the UK study, and patient data were captured from a variety of clinical settings (unlike our patient population, which only attended a wound care center). Therefore, having a “healthier” patient population in terms of the number of comorbidities is understandable. Nevertheless, the patients in the UK study were still very sick, and authors did report that many patients with wounds and diabetes also had musculoskeletal disorders (61%) and depression and/or anxiety (38%),^52^ which would have negatively impacted their QoL, independent of their wound status.

Another analysis of 1,000 patients with chronic leg ulcers in 10 wound care centers in Germany found similar comorbidity data to our study, with most patients having hypertension (70.5%); 27.2% having diabetes; and 24.4% having dyslipidemia (similar to our patients, of whom 28.8% had hyperlipidemia/pure hypercholesterolemia).^100^ A major difference in the German study was that nearly half of the population was morbidly obese with a Body Mass Index ≥30 (45.2%); while only 16.8% of patients in our study had this condition. The heterogeneous diversity of populations with chronic wounds cannot be understated, but the evidence consistently demonstrates that patients with wounds have multiple, serious comorbidities and multimorbidities.

The more comorbidities the patient has, especially those that have a huge impact on the patient, the more challenging it becomes to determine what the “true” utility value is for a given patient. For MAUIs, such as the EQ-5D-5L, derivation of utility values using the mapping process is a reflection, in part, of the country-specific culture and how individuals value different health-related issues.^3^ Methods designed to directly elicit utility values for aspects of single diseases suffer from several analogous limitations, as well as methodological issues, particularly for certain comorbidities whose impact varies, such as the diurnal pattern of rheumatoid arthritis,^101^ episodes of depression,^102^ or of a limited duration, such as surgical wounds.^9^ Consequently, while these values are certainly very useful, the methodology underpinning their estimation should still be judged as a “work in progress,” despite a positive future outlook.^33^

Early Canadian work suggested that for nonsevere comorbidities, using the multiplicative approach to calculate single utility values from two or three separate values associated with the comorbidities could lead to intuitive results.^103^ The work of Thompson et al. is probably state-of-the-art and was based on 929,565 individuals in United Kingdom, of which 30.5% had at least 2 conditions out of a possible 16.^99^

They calculated health state utility values for up to four joint health conditions (JHCs). Their findings suggest that the multiplicative approach was the best nonparametric estimator for two JHCs, but all the approaches, except for the linear parametric estimator, had substantial errors in the case of four JHCs. Our results showed considerable variation based on the four methods; we were unable to use a parametric method because this would have required a massive number of regression sets with too few samples in many of them due to the very large number of JHC permutations.

When we compare our health utility values to those derived from the EQ-5D for wound care patients reported in the literature (Table 5; median: 0.59 and mean: 0.56), we find that the mean and median *minimum* values of our study set (0.50 and 0.52, respectively) are in relatively good agreement. However, an issue with studying the utility of wound care populations is its variance. For example, we found that, while the median utility value for a DFU was 0.65, it decreased to 0.49 if at least two comorbidities were present.

**Table 5.** Health utility values for studies involving patients with any type of chronic wound derived from the EuroQol 5 Dimension 5

Study	EQ-5D Details	Wounds	Population	Health Utility
Tennvall et al. (2000)^104^	EQ-5D-3L, UK tariff	DFUs	Swedish adults	0.44
Morgan et al. (2006)^105^	EQ-5D-3L, NR	DFUs^a^	UK adults	0.29–0.51^b^
Michaels et al. (2009)^106^	EQ-5D-3L, UK tariff	VLUs	UK adults	0.65
Soares et al. (2009)^107^	EQ-5D-3L, TTO	VLUs	UK adults	0.50
Chuang et al. (2011)^108^	EQ-5D-3L, UK tariff	VLUs	UK adults	0.52
Padula et al. (2011)^109^	EQ-5D-3L, NR	PIs	American adults	0.6
Javanbakht et al. (2012)^110^	EQ-5D-3L, UK VAS tariff	DFUs^a^	Iranian adults	0.62
Siersma et al. (2017, 2013)^111,112^	EQ-5D-3L, NR	DFUs	European adults	0.65
Sobol et al. (2013)^113^	EQ-5D-3L, Polish tariff	DFUs	Polish adults	0.62
Li et al. (2017)^114^	EQ-5D-3L, Canadian tariff	DFUs	Canadian adults	0.59
Sothornwit et al. (2018)^115^	EQ-5D-5L, TTO	DFUs	Thai adults	0.70
Selva-Sevilla et al. (2020)^116^	EQ-5D-3L, Spanish tariff	Chronic wounds	Spanish adults	0.55
Arab-Zozani et al. (2022)^65^	EQ-5D-5L, cTTO	DFUs	Iranian adults	0.55

^a^ Only small percentages had DFUs.

^b^ Depending on type of diabetic comorbidities present.

cTTO, composite time trade-off; EQ-5D, EuroQol 5 Dimension 5; EQ-5D-3L, European Quality of Life 5 Dimensions 3 Level Version; EQ-5D-5L, European Quality of Life 5 Dimensions 5 Level Version; NR, not reported; TTO, time trade-off; VAS, Visual Analog Scale.

Our interpretation is that, if you try and ask the patient how they “value” this kind of wound, the more comorbidities the patient has, the lower the utility value. In other words, in the patient's world, it may not be possible to separate out the wound from the other comorbidities. This may, in part, also explain the variance of utility values in Table 5, although the severity of comorbidities in any given wound care population will also have a major effect. Consequently, given that the patients in our population may be sicker than the majority of those in Table 5 (meaning they would have lower utility values), the minimum approach seems a reasonable compromise.

This would mean that when looking at QoL from a utility scale for any patient, the comorbidity associated with the lowest utility value is the one that could most influence their QoL. Further studies are needed to confirm this concept. On the other hand, all other literature-reported approaches (additive, multiplicative, and ADE) are substantially lower, while the linear index method is too complex an undertaking for the huge number of comorbidity combinations, suggesting that these approaches have limitations once more than a few comorbidities used to calculate the health utility value.

In recent years, the W-QoL instrument was refined to make it shorter and easier to use by patients,^45^ but the elephant in the room, the influence of wound comorbidities on QoL, remains unaddressed. In our real-world analysis, it is concerning that the majority of patients (nearly 60%) had conditions that were worse than the utility values of their wounds. This means that their QoL was likely already quite low before their wound developed; for example, 8% of patients were bedridden in our study, which is a state close to death at a value of 0.13 (Table 2).

Many comorbid conditions captured by this analysis negatively impact a patient's mental health, restrict their activities and independence, and cause pain and/or sleep disturbance. Table 4 clearly shows how many comorbidities could potentially impact 10 of the 14 W-QoL items regardless of wound healing status. It should be emphasized that wounds still negatively affect the domains captured by the W-QoL and could make patients more depressed, have greater pain, and have more trouble sleeping and moving around. However, the real-world practicality of this questionnaire is limited, because it does not effectively, specifically capture wound impact, independent of the patient's general health status.

What does this mean for clinical practice? Teasing out the chronic wound contribution to the overall patient's QoL may in principle be useful, but quite limited for complex patients. As an example, consider a patient in a wheelchair with an autoimmune disease, a cardiovascular condition, and diabetes; they may likely develop a chronic wound as a consequence of their overall health status.

As wound care experts, we place the wound at the center of our concerns, but such wounds are more often a consequence of other conditions and not a root cause later affected by other conditions. In addition, many patients have multiple wounds. Thus, among patients with serious comorbid conditions, it may be unrealistic to expect a “wound-specific” QoL to be capable of measuring meaningful changes in response to the outcome of a specific wound. The point is that the best QoL tool should not target very specific conditions (such as wounds), but use a more realistic, holistic approach. Alternatively, additional questions regarding wounds should be added to the tools used to calculate health utility values based on various comorbid conditions.

In wound care research, W-QoL scores are used as surrogate endpoints for healing.^117^ Randomized, controlled trials tend to exclude patients with major comorbidities,^118^ and so, the trial design is not accurately capturing the general health state of the real-world patient population in wound care. Thus, practical limitations of the wound-centric QoL instrument hold true in trial settings, and current W-QoL assessments are an inappropriate use of time and resource utilization.

This study additionally provides proof of concept that even elderly patients with serious diseases (Fig. 1) can successfully utilize an electronic tablet to complete a standardized questionnaire, and that the results can be electronically transmitted to a quality registry where they can be seamlessly integrated into the patient's EHR. Unfortunately, a year after the completion of this study, CMS rejected the USWR-developed quality measure focused on the assessment of QoL among patients with chronic wounds. CMS officials stated that merely evaluating QoL was insufficient as a physician “quality activity” and that for a QoL process to be a valuable part of clinical care, it would be necessary to demonstrate a specific, measurable improvement in QoL in response to wound care.

Given the overriding impact of underlying disease on QoL, it will be difficult to develop a QoL assessment for chronic wound patients, which meets the CMS requirement. Since the measurement of QoL is time consuming to perform, expensive to implement, and wholly uncompensated, it is not likely to become part of routine clinical care among patients with chronic wounds. These findings do serve to emphasize the extreme level of illness among typical outpatients with chronic, nonhealing wounds.

### Limitations

There are several limitations to our analysis. First, we did not analyze the methods (nor their limitations) used in the literature to calculate utility values for each comorbidity. Second, we do not know how many wounds were involved in this convenience sample, because patients with multiple wounds did not have the specific number of wounds indicated; rather “multiple” was indicated. As this was a patient analysis, not a wound analysis, it is possible that large numbers of wounds could have affected the results.

Next, we did not know the severity of the wounds or conditions. For example, authors of a systematic review of 12 studies analyzing HR-QoL and DFU characteristics found an association between severity of the wound using the Wagner scale was also a significant predictor of overall HR-QoL in one study and social functioning in another study.^119^ The data used to analyze comorbidities from which utility values were calculated are quite old, and it is possible that the nature of the comorbidities might have changed. Finally, lack of data on the severity of comorbid conditions is also a limitation, as the severity of a condition plays an important role in the HR-QoL.

## FUTURE DIRECTIONS

More work is needed to create a PROM that can measure response to treatment among patients with chronic wounds.

## SUMMARY

Despite the popularity of PROMs, it will be difficult to find a useful role for them in day-to-day wound management. In a large proportion of cases, it is likely that QoL may be dominated by one or two major underlying medical conditions, which impact the patient even more than their chronic wound(s).

**TAKE-HOME MESSAGES**

- Patients with chronic wounds have many comorbidities, some of which may dominate a patient's life and perception of QoL, more so than their chronic wounds.
- While the W-QoL instrument is a useful questionnaire, clinicians should be aware that some medical conditions could unduly influence scores.
- The QoL of patients as measured by the health utilities index (HUI)—a single score—is low, meaning that most individuals rate their QoL as poor.

## ACKNOWLEDGMENTS AND FUNDING SOURCES

The authors would like to acknowledge the dedicated efforts of Melissa J. Anders, BSN, CWOCN, and Debi Thompson, BSN, in obtaining the W-QoL assessments from patients whose data were analyzed in this study. Strategic Solutions, Inc., funded this study. The USWR funded the CMS-approved quality measure initiative, the electronic tablets that were programmed with the W-QoL, and provided the data for analysis.

## Supplementary Material

Supplemental data

Supplemental data

Supplemental data

Supplemental data

## ABOUT THE AUTHORS

**Kristen A. Eckert** is a certified Medical Writer and Editor with more than 16 years of experience working with health care. Ms. Eckert previously completed her graduate studies at the University of Cambridge. As the medical writer, she has contributed to over 100 peer-reviewed medical publications in wound care and other pathologies, and was also author and investigator to at least a third of these publications. **Caroline E. Fife, MD,** did her residency in Family Medicine at the University of Texas, Southwestern in Dallas, followed by a Fellowship in Undersea and Hyperbaric Medicine (UHM) at Duke University. A Certified Wound Specialist since 1998, she is now the Chief Medical Officer of Intellicure, the Executive Director of the USWR, and a Professor of Geriatrics at Baylor College of Medicine in Houston. **Marissa J. Carter** has an **MA** in biochemistry from Oxford University and a **PhD** in chemistry from Brandeis University. She is an evidence-based medicine reviewer, biostatistician, and epidemiologist, and is President of Strategic Solutions, a company that designs, assists with, and analyzes clinical trials. She has also been active with WHO and FDA initiatives, and currently chairs a clinical trial reporting group with the Wound Care Collaborative Community (WCCC).

## Abbreviations and Acronyms

ADE: adjusted decrement estimator
BKA: below-knee amputations
CEAR: Cost-Effectiveness Analysis Registry
CMS: The Centers for Medicare and Medicaid Services
COPD: chronic obstructive pulmonary disease
cTTO: composite time trade-off
DCE: discrete choice experiment
DFU: diabetic foot ulcer
EHR: electronic health record
HR-QoL: health-related quality of life
HUI: health utilities index
IRB: Institutional Review Board
JHC: joint health condition
MAUI: multiattribute utility instrument
NR: not reported
PBM: preference-based measure
PI: pressure injury
PROM: patient-reported outcome measure
PTSD: posttraumatic stress disorder
QoL: quality of life
SD: standard deviation
SF: short form
SG: standard gamble
SSI: surgical site infection
TTO: time trade-off
USWR: U.S. Wound Registry
VAS: Visual Analog Scale
VLU: venous leg ulcer
W-QoL: Wound-Quality of Life
