# Supplementary material for: The Impact of Underlying Conditions on Quality-of-Life Measurement Among Patients with Chronic Wounds, as Measured by Utility Values: A Review with an Additional Study
Source: Adv Wound Care (New Rochelle). 2023 Oct 19;12(12):680–95. doi: 10.1089/wound.2023.0098 (PMC10615090; doi:10.1089/wound.2023.0098)
Supplement: Supplemental data [file Suppl_TableS1.docx]

Supplemental Table 1. Comorbid conditions without utility values (n = 33)

| **Category** | **Condition** |
| --- | --- |
| Autoimmune | Primary biliary cirrhosis |
|  | Systemic lupus erythematous |
|  | CREST syndrome |
|  | Lupus (undifferentiated tissue syndrome) |
|  | Sjogrens syndrome |
| Cardiovascular/Vascular | Raynauds Syndrome without gangrene |
|  | Arnold Chiari brain malformation |
|  | Churg Strauss Syndrome |
| Hematological Conditions/ Coagulopathies | Polycythemia rubra vera |
|  | Coagulopathy |
|  | Inferior vena cava filter for pulmonary embolism |
|  | Porphyria cuttanea tarda |
|  | Antiphospholipid antibody |
|  | Factor 5 Leiden |
|  | Hemochromatosis |
| Metabolic/Endocrinological | Hyperglycemia |
|  | Addisons disease |
|  | Ovarian failure |
|  | Disorders of calcium metabolism |
|  | Adrenal insufficiency |
| Neurological | Menieres disease |
| Nutritional | Vitamin D deficiency |
|  | Protein-calorie malnutrition (including mild/severe) |
|  | Vitamin B12 deficiency |
|  | Malnutrition – severe underweight |
|  | Anasarca (starvation/severe malnutrition) |
| Orthopedic/Musculoskeletal | Hand contractures |
|  | Myelofibrosis |
|  | Severe kyphosis |
|  | Thoracic outlet syndrome |
|  | Compartment Syndrome |
| Social Determinants of Health | Alcohol/drug dependency |
| Urinary/Renal | Focal Sclerosing Glomerulonephritis |
