# Supplementary material for: The Impact of Underlying Conditions on Quality-of-Life Measurement Among Patients with Chronic Wounds, as Measured by Utility Values: A Review with an Additional Study
Source: Adv Wound Care (New Rochelle). 2023 Oct 19;12(12):680–95. doi: 10.1089/wound.2023.0098 (PMC10615090; doi:10.1089/wound.2023.0098)
Supplement: Supplemental data [file Suppl_TableS2.docx]

**Supplemental Table 2.** Proxy utility values imputed for comorbid conditions missing utility values (n = 81)

| **Proxy Condition Used for Utility Value** | **Conditions Missing Utility Values** |
| --- | --- |
| Abnormalities of gait and mobility | Multiple back surgeries; slipping, tripping, and stumbling without falling; avascular necrosis in shoulders, bilateral; Myasthenia Gravis; muscle contractures, bilateral lower extremities; Charcot’s deformity; myotonic dystrophy; transverse myelitis |
| Aortic valve replacement | Transaortic valve repair |
| Asthma | Asthmatic bronchitis |
| Attention Deficit Hyperactivity Disorder | Attention Deficit Disorder |
| Bedridden | Functional quadriplegia |
| Bleeding ulcer | Chronic or unspecified gastric ulcer with hemorrhage history |
| Cellulitis | Hidradenitis suppurativa; bullous pemphigoid |
| Complicated/nonhealing wound | Burns (no degree indicated); crushing injury; chronic ulcer related to lymphedema; chronic wound related to prurigo; ulcer related to cancer; toe ulcer related to malnutrition; ulcer related to atrophie blanche on foot; leg ulcer related to chronic liver disease; chronic wound related to neuropathy |
| Chronic obstructive pulmonary disease | Chronic airway obstruction |
| Chronic pelvic pain syndrome | Prostatitis |
| Chronic venous insufficiency | Vein surgery bilateral lower extremities |
| Deep vein thrombosis | History of deep vein thrombosis and/or bilateral; history of blood clot in left leg |
| Degenerative joint disease | Back pain from degenerative disc disease |
| Diabetes + complications | Diabetes with other specified manifestations, type 1 |
| Diabetes + peripheral arterial disease | (Diabetic) peripheral angiopathy |
| Diabetic nephropathy | Type 2 diabetes with other diabetic kidney complication |
| Diabetic neuropathy | Diabetes with neurological manifestations, type 2 |
| Falls | Right femur fracture due to fall |
| Finger amputation | Multiple finger amputations |
| Hip replacement | Bilateral hip replacement |
| History of endometrial cancer | History of malignant neoplasm of other parts of uterus |
| History of leukemia | History of multiple myeloma |
| History of lung cancer | History of other malignant neoplasm of bronchus and lung |
| Hyperlipidemia | Pure hypercholesterolemia |
| Hyperthyroidism | Graves disease |
| Inflammatory arthropathy | Diabetic arthropathy |
| Knee meniscus repair | Bilateral knee meniscus repair |
| Knee replacement | Bilateral knee replacement |
| Mild Alzheimers disease | Mild cognitive impairment on Aricept |
| Minor amputation | Left ear removal due to cancer and radiation |
| Necrosis | Third degree burns; polyarteritis nodosa; giant cell temporal arteritis, gangrene, pyoderma gangrenosum, arteritis, Raynauds syndrome, systemic sclerosis/scleroderma, necrobiosis lipoidica, necrotizing fasciitis, vasculitis |
| Neuropathy | Nerve pain, polyneuropathy due to other toxic agents |
| Oropharyngeal cancer | Squamous cell carcinoma nasopharyngeal/right tonsil |
| Open reduction and internal fixation | Bimalleolar/ankle fracture, closed |
| Opioid use for pain | On narcotic pain medication, chronic pain syndrome |
| Ostomy | Urinary ostomy |
| Peripheral arterial disease | Atherosclerosis of native arteries of the extremities with ulceration/arterial ulcer, type 2 diabetes with ulcer due to calcinosis cutis, transient ischemic attack |
| Pituitary replacement therapy | Pituitary tumor and gland removal |
| Postgastrointestinal bleeding | History of gastric bleeding from anticoagulation |
| Postpacemaker | Syncope |
| Postpulmonary embolism | History of blood clots in lung |
| Postthyroidectomy | History of malignant neoplasm of thyroid |
| Primary/essential hypertension | Malignant essential hypertension |
| Rheumatoid arthritis | Unknown connective tissue disorder |
| Rutherford 4 (moderate: peripheral arterial disease)^a^ | Thromboangiitis obliterans (Buerger’s disease) |
| Schizophrenia | Schizoaffective disorder |
| Sciatica | Lumbar radiculopathy |
| Severe disability after neck surgery | Neck surgery |
| Spinal fusion | Cervical spine surgery - 3 levels |
| Wheelchair status | Bilateral leg amputations |

^a^Lowest utility available for peripheral arterial disease to capture the severity of the condition
