# Supplementary material for: The Impact of Underlying Conditions on Quality-of-Life Measurement Among Patients with Chronic Wounds, as Measured by Utility Values: A Review with an Additional Study
Source: Adv Wound Care (New Rochelle). 2023 Oct 19;12(12):680–95. doi: 10.1089/wound.2023.0098 (PMC10615090; doi:10.1089/wound.2023.0098)
Supplement: Supplemental data [file Suppl_TableS3.docx]

**Supplemental Table 3.** Classification of comorbid conditions with their median utility values, including those with imputed proxy values (n = 352).

| **Category** | **Condition** | **Median Utility Value** |
| --- | --- | --- |
| Ambulatory Status | Abnormalities of gait and mobility | 0.7 |
|  | Bedridden/bed confinement status | 0.13 |
|  | Fall(s), history of fall(s) | 0.44 |
|  | Gait disturbance(s) | 0.7 |
|  | In a wheelchair | 0.35 |
|  | Slipping, tripping, stumbling, without falling | 0.7 |
|  | Unsteadiness on feet | 0.67 |
| Autoimmune | Alopecia universalis | 0.67 |
|  | Celiac disease | 0.84 |
|  | Chronic immune suppression, with/without prednisone | 0.57 |
|  | Giant cell temporal arteritis | 0.42 |
|  | Graves disease | 0.95 |
|  | Guillain-Barre Syndrome | 0.4 |
|  | Psoriasis | 0.81 |
|  | Psoriatic arthritis | 0.63 |
|  | Psoriatic arthropathy | 0.63 |
|  | Rheumatoid arthritis | 0.69 |
|  | Systemic scleroderma with/without ulcer | 0.42 |
|  | Unknown connective tissue disorder | 0.69 |
| Cardiovascular/ Vascular | Aorta graft for aortic aneurysm | 0.72 |
|  | Aorta stenosis regurgitation (mild) | 0.75 |
|  | Aortic sclerosis | 0.64 |
|  | Aortic stenosis, post | 0.75 |
|  | Aortic valve replacement/transaortic valve repair | 0.74 |
|  | Acute myocardial infarction(s) | 0.77 |
|  | Acute myocardial infarction status post coronary artery bypass graft | 0.84 |
|  | Angina | 0.77 |
|  | Angioplasty, unilateral/bilateral | 0.87 |
|  | Arrhythmia | 0.98 |
|  | Arterial ulcer(s) | 0.64 |
|  | Arteritis | 0.42 |
|  | Asymptomatic varicose veins | 0.78 |
|  | Atherosclerosis of native arteries of extremities, unilateral/bilateral/unspecified | 0.64 |
|  | Atherosclerosis of native arteries of extremities with intermittent claudication | 0.71 |
|  | Atherosclerotic heart disease | 0.87 |
|  | Atrial fibrillation | 0.76 |
|  | Atrial fibrillation with prior stroke | 0.67 |
|  | Avascular necrosis | 0.7 |
|  | Cardiomyopathy, ischemic/nonischemic | 0.8 |
|  | Carotid endarterectomy | 0.71 |
|  | Chronic systolic heart failure with left ventricular assist device implanted | 0.72 |
|  | Chronic venous hypertension | 0.96 |
|  | Chronic venous insufficiency | 0.96 |
|  | Chronic venous ulcer(s) | 0.69 |
|  | Congestive heart failure | 0.66 |
|  | Coronary artery bypass graft(s) | 0.84 |
|  | Coronary artery disease | 0.82 |
|  | Cerebrovascular disease | 0.65 |
|  | Deep vein thrombosis, chronic | 0.57 |
|  | Deep vein thrombosis, unilateral/bilateral, history/presence of | 0.80 |
| Cardiovascular/  Vascular *continued* | Defibrillator | 0.86 |
|  | Endocarditis | 0.58 |
|  | Femoral-popliteal bypass | 0.94 |
|  | Heart murmur with mitral valve leak | 0.82 |
|  | Heart transplant | 0.77 |
|  | Hemiplegia from stroke | 0.84 |
|  | History of stroke(s) | 0.61 |
|  | Hypertension, cardiopulmonary | 0.63 |
|  | Hypertension, primary (essential), benign/unspecified/malignant | 0.78 |
|  | Hypertension, pulmonary | 0.66 |
|  | Mitral regurgitation (severe) | 0.66 |
|  | Mitral valve replacement | 0.88 |
|  | Occlusion and stenosis of unspecified cerebral artery | 0.65 |
|  | Pacemaker | 0.81 |
|  | Peripheral arterial disease | 0.64 |
|  | Polyarteritis nodosa | 0.42 |
|  | Postphlebitic syndrome | 0.93 |
|  | Postpulmonary embolism/history of clots in lung | 0.95 |
|  | Postthrombotic syndrome | 0.93 |
|  | Pulmonary embolus | 0.75 |
|  | Rapid heart rate | 0.83 |
|  | Raynauds disease | 0.42 |
|  | Sick sinus syndrome | 0.78 |
|  | Stent(s) | 0.94 |
|  | Supraventricular tachycardia | 0.83 |
|  | Syncope (postpacemaker) | 0.76 |
|  | Thomboangiitis obliterans | 0.49 |
|  | Transient ischemic attack | 0.64 |
|  | Varicose veins with ulceration | 0.69 |
|  | Vasculitis, angiitisis, arteritis | 0.42 |
|  | Vein surgery | 0.96 |
|  | Venous ablation, bilateral | 0.81 |
| Digestive | Alcoholic cirrhosis without ascites | 0.75 |
|  | Ascites | 0.52 |
|  | Cholecystectomy | 0.9 |
|  | Chronic liver disease | 0.22 |
|  | Chronic or unspecified gastric ulcer with hemorrhage history | 0.46 |
|  | Cirrhosis of liver | 0.77 |
|  | Colon fistula | 0.48 |
|  | Colon resection | 0.82 |
|  | Colostomy | 0.82 |
|  | Crohns disease | 0.77 |
|  | Diarrhea with/without constipation | 0.74 |
|  | Encephalopathy (hepatic) | 0.46 |
|  | Fecal incontinence | 0.68 |
|  | Gastric bypass | 0.74 |
|  | Gastroesophageal reflux disease | 0.76 |
|  | History of gastrointestinal bleeding | 0.98 |
|  | History of bleeding stomach ulcers | 0.46 |
|  | Irritable bowel syndrome | 0.74 |
|  | Liver failure | 0.8 |
|  | Nonalcoholic fatty liver disease | 0.84 |
|  | Peg feeding tube | 0.85 |
| Ear/Vestibular | Dizziness/vertigo  Hearing loss with hearing aids | 0.9  0.82 |
| Hematological Conditions/ | Anticoagulation | 0.99 |
|  | Hyperlipidemia | 0.89 |
| Coagulopathies | Hypoglycemia without coma | 0.75 |
|  | History of gastric bleeding from anticoagulation | 0.98 |
|  | History of sepsis | 0.64 |
|  | Myelodysplastic syndrome | 0.66 |
|  | Prothrombin factor 2 | 0.99 |
|  | Pure hypercholesterolemia | 0.89 |
| Infectious Disease | Chronic Hepatitis C without mention of hepatic coma | 0.82 |
|  | Chronic liver disease/hepatitis | 0.6 |
|  | Herpes zoster | 0.77 |
|  | History of polio with postpolio syndrome | 0.34 |
|  | History of tuberculosis | 0.95 |
|  | Monoplegia from polio | 0.37 |
| Mental | Attention Deficit Disorder | 0.84 |
|  | Attention Deficit Hyperactivity Disorder | 0.84 |
|  | Anxiety | 0.65 |
|  | Bipolar disorder/mania | 0.69 |
|  | Depression/depressive disorder | 0.6 |
|  | Major depressive disorder | 0.54 |
|  | Panic attack | 0.65 |
|  | Posttraumatic Stress Disorder | 0.54 |
|  | Schizoaffective disorder/schizophrenia | 0.66 |
|  | Suicide attempt | 0.53 |
| Metabolic/  Endocrinological | Acromegaly | 0.73 |
|  | Diabetes, insulin-dependent | 0.84 |
|  | Diabetes, juvenile Type 1 | 0.83 |
|  | Diabetes, Type 2/unspecified^a^ | 0.79 |
|  | Diabetes with complications/other specified manifestations^a^ | 0.77 |
|  | Diabetes, uncontrolled | 0.77 |
|  | Diabetes with hypoglycemia | 0.75 |
|  | Diabetic (peripheral) angiopathy | 0.64 |
|  | Diabetic arthropathy | 0.69 |
|  | Diabetic foot ulcer | 0.65 |
|  | Diabetic foot ulcer with at least 2 comorbidities | 0.49 |
|  | Diabetes with major amputation | 0.61 |
|  | Diabetes with minor amputation | 0.68 |
|  | Diabetes with transmetatarsal amputation | 0.95 |
|  | Diabetic nephropathy/kidney complications | 0.68 |
|  | Diabetic (peripheral autonomic/poly-) neuropathy | 0.48 |
|  | Diabetic retinopathy | 0.73 |
|  | Diabetic retinopathy with unilateral blindness | 0.65 |
|  | Diabetic ulcer on amputation stump | 0.45 |
|  | Diabetic ulcer (not on foot) | 0.68 |
|  | Hyperthyroidism | 0.95 |
|  | Hypothyroidism | 0.9 |
| Neoplasms/ | Active malignant neoplasm of bronchus and lung | 0.65 |
| Cancers | Active malignant neoplasm of soft tissue, likely sarcoma | 0.51 |
|  | Active multiple myeloma or not having achieved remission | 0.7 |
|  | Active pancreatic cancer | 0.72 |
|  | Carcinoid syndrome | 0.79 |
|  | Chronic lymphocytic leukemia | 0.69 |
| Neoplasms/  Cancers *continued* | Chronic ulcer related to cancer | 0.61 |
|  | Colon cancer with metastasis | 0.65 |
|  | Current chemotherapy treatments | 0.64 |
|  | Dehydration and diarrhea from chemotherapy | 0.61 |
|  | History of basal cell carcinoma removal/squamous cell removal | 1 |
|  | History of bladder cancer | 0.96 |
|  | History of bone cancer | 0.85 |
|  | History of breast cancer | 0.85 |
|  | History of bronchus and lung cancer | 0.84 |
|  | History of colon cancer with colectomy | 0.82 |
|  | History of esophageal cancer | 0.84 |
|  | History of female genital organs | 0.76 |
|  | History of hepatocellular carcinoma | 0.78 |
|  | History of intestinal cancer | 0.89 |
|  | History of leukemia/lymphoid leukemia | 0.93 |
|  | History of lip, oral cavity, and phaynyx cancer | 0.78 |
|  | History of lymphoma | 0.83 |
|  | History of malignant melanoma | 0.8 |
|  | History of metastatic colorectal cancer | 0.73 |
|  | History of multiple myeloma | 0.93 |
|  | History of other skin cancer | 1 |
|  | History of ovarian cancer | 0.78 |
|  | History of prostate cancer | 0.85 |
|  | History of rectal cancer | 0.73 |
|  | History of thyroid cancer, postthydroidectomy | 0.97 |
|  | History of uterine cancer with hysterectomy | 0.8 |
|  | History of uterine cancer without hysterectomy | 0.88 |
|  | Left ear removal due to cancer and radiation | 0.69 |
|  | Lumpectomy with node resection and radiation | 0.89 |
|  | Mastectomy, bilateral | 0.88 |
|  | Mastectomy, unilateral | 0.91 |
|  | Mastectomy with reconstruction | 0.84 |
|  | Melanoma removal | 0.8 |
|  | Pituitary tumor with/without gland removal | 0.75 |
|  | Radiation complication of graft | 0.72 |
|  | Radiation necrosis | 0.74 |
|  | Squamous cell carcinoma nasopharyngeal | 0.58 |
|  | Wound from radiation + radical neck dissection for sweat gland cancer | 0.68 |
| Neurological | Alzheimers disease (mild) | 0.6 |
|  | Chronic neuropathic pain | 0.56 |
|  | Chronic neuropathic/polyneuropathic ulcer | 0.61 |
|  | Closed head injury | 0.89 |
|  | Cognitive impairment (mild) | 0.6 |
|  | Dementia (mild) with/without behavioral disturbance | 0.68 |
|  | Dystonia | 0.76 |
|  | Epilepsy | 0.8 |
|  | Foot drop | 0.7 |
|  | Functional quadriplegia | 0.13 |
|  | Movement disorder in legs | 0.64 |
|  | Multiple sclerosis | 0.62 |
|  | Myasthenia Gravis | 0.7 |
|  | Nerve pain | 0.62 |
|  | Neuropathy, chronic demyelinating idiopathic | 0.62 |
|  | Neuropathy, hereditary/idiopathic peripheral/autonomic/other | 0.62 |
|  | Parkinsons disease | 0.6 |
|  | Polyneuropathy, idiopathic progressive/drug-induced/toxic agents | 0.62 |
| Neurological *continued* | Quadriplegia | 0.39 |
|  | Restless leg syndrome | 0.54 |
|  | Sciatic nerve impingement | 0.76 |
|  | Seizures | 0.75 |
|  | Spinal cord implant for pain stimulation system | 0.62 |
|  | Spinal cord injury | 0.5 |
|  | Stereotactic surgery | 0.81 |
|  | Transverse myelitis | 0.7 |
|  | Traumatic brain injury | 0.89 |
|  | Tremors | 0.8 |
| Nutritional | Anemia with/without transfusions | 0.75 |
|  | Anemia of chronic kidney disease | 0.55 |
|  | Malnutrition, severe underweight, BMI <18 | 0.74 |
|  | Morbid, severe obesity | 0.75 |
|  | Obesity | 0.79 |
|  | Overweight | 0.82 |
| Orthopedic/  Musculoskeletal  Orthopedic/ Musculoskeletal *continued* | Arthritis | 0.69 |
|  | Stenosis in lumbar spine | 0.57 |
|  | Back surgery with disc replacement/repair rods placed | 0.57 |
|  | Back surgery, lumbar/spinal fusion, multiple | 0.59 |
|  | Baclofen pump for muscle spasms | 0.66 |
|  | Cervical/lumbar laminectomy | 0.57 |
|  | Cervical fusion | 0.7 |
|  | Cervicalgia/neck pain | 0.7 |
|  | Charcot foot/deformity | 0.7 |
|  | Chronic back pain | 0.64 |
|  | Chronic fatigue | 0.57 |
|  | Chronic pain, unspecified | 0.56 |
|  | Chronic pain on opioids/chronic opioid use/nonopioids medications/narcotics/ morphine/muscle relaxants | 0.56 |
|  | Degenerative disc disease, back | 0.5 |
|  | Drop foot | 0.7 |
|  | Fibromyalgia | 0.55 |
|  | Generalized weakness | 0.29 |
|  | Gout | 0.71 |
|  | Hemilaminectomy, medial facetectomy, and foraminotomy | 0.59 |
|  | Herniated disc | 0.56 |
|  | Hip fracture(s), prior | 0.68 |
|  | Hip prothesis dislocation with revision | 0.98 |
|  | Hip replacement, bilateral/unilateral | 0.77 |
|  | Inflammatory polyarthropathy | 0.69 |
|  | Knee arthroplasty | 0.84 |
|  | Knee meniscus repair, bilateral/unilateral | 0.78 |
|  | Knee replacement, bilateral/unilateral | 0.84 |
|  | Kyphoplasty | 0.28 |
|  | Lumbar radiculopathy | 0.76 |
|  | Muscle atrophy | 0.81 |
|  | Muscle contractures | 0.7 |
|  | Myotonic dystrophy | 0.7 |
|  | Myalgia | 0.85 |
|  | Neck surgery | 0.61 |
|  | Open Reduction Internal Fixation, ankle, prior ankle fracture | 0.7 |
|  | Open Reduction Internal Fixation, hip/pelvic fracture | 0.68 |
|  | Osteoarthritis, primary | 0.64 |
|  | Osteonecrosis | 0.67 |
|  | Osteoporosis with fracture | 0.81 |
|  | Osteoporosis without current pathological fracture | 0.75 |
|  | Reconstruction after replacement following knee arthroplasty | 0.58 |
|  | Resection | 0.7 |
|  | Rotator cuff repair(s) | 0.85 |
|  | Scoliosis | 0.7 |
|  | Scoliosis correction surgery | 0.58 |
|  | Shoulder and hip fractures | 0.68 |
|  | Shoulder replacement, bilateral/unilateral | 0.9 |
|  | Slipping, tripping, stumbling, without falling | 0.7 |
|  | Spondylosis, cervical | 0.7 |
| Respiratory | Sleep apnea | 0.81 |
|  | Asthma | 0.79 |
|  | Asthma, cough variant | 0.79 |
|  | Asthmatic bronchitis | 0.79 |
|  | Chronic airway obstruction | 0.73 |
|  | Chronic bronchitis | 0.75 |
|  | Chronic obstructive pulmonary disease | 0.73 |
|  | Dependence on supplemental oxygen | 0.45 |
|  | Emphysema | 0.68 |
|  | Interstitial lung disease | 0.74 |
|  | Pulmonary edema, flash/recurrent | 0.7 |
|  | Shortness of breath with/without fluid retention | 0.62 |
| Reproductive | Benign prostatic hypertrophy | 0.9 |
|  | Erectile dysfunction | 0.87 |
|  | Prostatitis | 0.6 |
| Urinary/Renal | Acute kidney/renal failure | 0.62 |
|  | Bladder/urinary incontinence | 0.74 |
|  | Chronic kidney disease | 0.86 |
|  | Chronic kidney disease on dialysis | 0.68 |
|  | Chronic kidney disease, stage 4 (severe) | 0.84 |
|  | Chronic urinary tract infection associated with catheter | 0.72 |
|  | End stage renal disease | 0.62 |
|  | Hypertensive chronic kidney disease | 0.67 |
|  | Kidney stent | 0.75 |
|  | Kidney transplant, unilateral/bilateral | 0.8 |
|  | Neurogenic bladder with/without urostomy | 0.61 |
|  | Neurogenic bowel | 0.68 |
|  | Polycystic kidney disease (on dialysis) | 0.62 |
|  | Stress urinary incontinence | 0.8 |
|  | Urinary ostomy | 0.78 |
| Visual Disorders | Age-related macular degeneration | 0.75 |
|  | Blindness, unilateral | 0.71 |
|  | Glaucoma | 0.87 |
|  | Legally blind | 0.48 |
|  | Macular degeneration | 0.69 |
|  | Macular degeneration with unilateral blindness | 0.58 |
| Wounds/ | Accident causing wound(s) | 0.7 |
| Dermatological | Amputation, finger(s) | 0.47 |
|  | Amputation, minor/toe(s) without diabetes | 0.69 |
|  | Major amputation without diabetes | 0.4 |
|  | Nonhealing amputation | 0.45 |
|  | Angioedema, recurrent | 0.67 |
|  | Atrophie blanche | 0.61 |
| Wounds/  Dermatological *continued* | Bullous pemphigoid | 0.6 |
|  | Burn, third degree | 0.42 |
|  | Burn, unspecified | 0.61 |
|  | Calcinosis cutis with ulcer | 0.64 |
|  | Carbuncle abscess | 0.64 |
|  | Cutaneous abscess | 0.64 |
|  | Cellulitis | 0.6 |
|  | Chronic/complicated wound(s)/ulcer(s) | 0.61 |
|  | Chronic ulcer due to gait and mobility | 0.61 |
|  | Crush injury | 0.61 |
|  | Dehiscence | 0.54 |
|  | Dermatitis | 0.76 |
|  | Edema, chronic/acute, localized/generalized, bilateral/unilateral | 0.9 |
|  | Flap | 0.4 |
|  | Gangrene | 0.42 |
|  | Hematoma, chronic | 0.64 |
|  | Hidradenitis suppurativa | 0.6 |
|  | Lymphedema, hereditary/nonhereditary/other | 0.8 |
|  | Lymphedema causing chronic ulcer | 0.61 |
|  | Necrobiosis lipoidica | 0.42 |
|  | Necrotizing fasciitis | 0.42 |
|  | Nonhealing/complicated surgical wound(s) | 0.52 |
|  | Open wound(s) | 0.7 |
|  | Osteomyelitis, acute/chronic, multiple | 0.64 |
|  | Pilonidal cyst | 0.64 |
|  | Postoperative fistula | 0.49 |
|  | Pressure injury(-ies) | 0.64 |
|  | Prurigo (chronic) | 0.61 |
|  | Pyoderma gangrenosum | 0.42 |
|  | Wound infection | 0.56 |
|  | Wound infection, on antibiotics | 0.46 |
|  | Wound infection from carpal tunnel surgery | 0.75 |

^a^Median utility value calculated from utility values obtained from literature registered on the Cost-Effectiveness Analysis Registry^6^ and from an article, Takahara et al, 2019,^54^ obtained from PubMed
