# Supplementary material for: The Impact of Underlying Conditions on Quality-of-Life Measurement Among Patients with Chronic Wounds, as Measured by Utility Values: A Review with an Additional Study
Source: Adv Wound Care (New Rochelle). 2023 Oct 19;12(12):680–95. doi: 10.1089/wound.2023.0098 (PMC10615090; doi:10.1089/wound.2023.0098)
Supplement: Supplemental data [file Suppl_TableS4.docx]

Supplemental Table 4. Utility values and descriptors used to calculate median utility values for wounds from the Cost-Effectiveness Analysis Registry and recent literature.^6^

| **Descriptors** | **Utility Values** | **Reference Number(s)** |
| --- | --- | --- |
| Diabetic foot ulcer, median value: 0.65  active  diabetic foot, superficial ulcer  no complications  diabetic foot, deep ulcer  uncomplicated, noninfected  ambulatory + diabetic foot ulcer  nonambulatory due to diabetic foot ulcer  diabetic foot ulcer in individuals with type 2 diabetes  diabetes mellitus with ongoing ulcer  uninfected/unhealed ulcer  recurred diabetic foot ulcer  diabetic foot ulcer, EQ-5D-5L  diabetic foot ulcer, cTTO | 0.62  0.75  0.88  0.75  0.75  0.73  0.65  0.59  0.44  0.47  0.47  0.55  0.67 | 55  56  57  56  57–59  60  60  61  62  63,64  64  65^a^  65^a^ |
| Diabetic foot ulcer with ≥2 comorbidities, median value: 0.49  Diabetic foot ulcer with at least 2 comorbidities, EQ-5D-5L  Diabetic foot ulcer with at least 2 comorbidities, cTTO | 0.45  0.53 | 65^a^  65^a^ |
| Venous leg ulcer, median value: 0.69 |  |  |
| venous leg ulcer | 0.64 | 66 |
| improving venous leg ulcer | 0.73 | 66 |
| Pressure injury, median value: 0.64 |  |  |
| pressure ulcer | 0.59 | 66 |
| improving pressure ulcer | 0.68 | 66 |
| pressure ulcer | 0.68 | 67 |
| single severe pressure ulcer | 0.36 | 68 |
| single superficial pressure ulcer/grade 1 or 2 | 0.68 | 68,69 |
| inflammation/senescence in pressure ulcer | 0.36 | 70 |
| Surgical wound/dehiscence median value, 0.52^b^ |  |  |
| wound dehiscence | 0.54 | 71,72 |
| unhealed (persistent/recurrent) foot wound or unhealed amputation stump wound,  surgical bypass | 0.5 | 73 |
| unhealed (persistent/recurrent) foot wound or unhealed amputation stump wound,  primary amputation | 0.48 | 73 |
| unhealed (persistent/recurrent) foot wound or unhealed amputation stump wound,  primary amputation | 0.42 | 73 |
| after 2 weeks, with complications | 0.67 | 9 |
| after 4 weeks, with complications | 0.7 | 9 |
| Finger amputations, median value: 0.47 |  |  |
| amputation of finger | 0.1 | 74 |
| single finger (not thumb) revision amputation | 0.84 | 75 |
| Major amputation without diabetes, median value: 0.4 |  |  |
| major amputation | 0.19 | 76 |
| major lower extremity amputation | 0.11 | 77 |
| major amputation | 0.7 | 78,79 |
| patients after the healing of a major amputation | 0.31 | 80 |
| major amputation from open surgical repair of abdominal aortic aneurysms | 0.8 | 81 |
| above-knee amputation | 0.48 | 82 |
| above knee amputation | 0.2 | 83,84 |
| above the knee amputation | 0.61 | 85 |
| Minor amputation without diabetes: 0.69 |  |  |
| Minor foot amputation | 0.69 | 59 |
| Diabetes + minor amputation, median value: 0.68 |  |  |
| diabetes + minor amputation | 0.72 | 58 |
| diabetic foot, post minor amputation | 0.68 | 86 |
| diabetes after healing with minor amputation | 0.61 | 62 |
| Diabetes with major amputation, median value: 0.61 |  |  |
| major amputation + diabetes, (EQ-5D) | 0.65 | 58 |
| diabetes mellitus (major amputation) | 0.62 | 86 |
| diabetes with lower extremity amputation/due to diabetes/type 2 diabetes/insulin-  dependent diabetes (TTO) | 0.8 | 87–91 |
| diabetes after healing with major amputation, (EQ-5D-VAS) | 0.31 | 62,92 |
| lower-extremity amputation, type 2 diabetes | 0.58 | 93 |
| diabetes, age >=65 years old, with lower-extremity amputation, TTO approach | 0.45 | 88 |
| below knee amputation in patients with type 2 noninsulin-dependent diabetes  mellitus (other indirect/generic measures) | 0.8 | 94 |
| above knee amputation in patients with type 2 noninsulin-dependent diabetes  mellitus (other indirect/generic measures) | 0.5 | 94 |
| Diabetes with transmetatarsal amputation: 0.95 |  |  |
| transmetatarsal amputation in patients with type 2 noninsulin-dependent diabetes  mellitus | 0.95 | 94 |
| Abscess median utility value: 0.64 |  |  |
| abscess | 0.64 | 95 |
| occurrence of an abscess | 0.65 | 96 |
| Uncomplicated open wound: 0.7 |  |  |
| outpatient treatment of open wound, infected, or both | 0.7 | 97 |
| Complicated wound: 0.61 |  |  |
| Wound complication | 0.61 | 98 |

^a^Reference not included in registry but found from PubMed literature search

^b^unhealed amputation has a median value of 0.45.

EQ-5D = EuroQOL 5 Dimensions Questionnaire; TTO = time trade-off; VAS = Visual Analogue Scale.
